# The prevalence and influencing factors of COVID-19 in pregnant women post-relaxation of epidemic control measures in Hunan Province, China

**DOI:** 10.3389/fmed.2025.1485157

**Published:** 2025-02-03

**Authors:** Yingxia Wang, Yixu Liu, Kehan Zou, Min Yang, Yinglan Wu, Donghua Xie

**Affiliations:** ^1^Department of Women Health Management, Hunan Provincial Maternal and Child Health Care Hospital, Changsha, Hunan, China; ^2^Xiangya School of Public Health, Central South University, Changsha, Hunan, China; ^3^Department of Information Management, Hunan Provincial Maternal and Child Health Care Hospital, Changsha, Hunan, China

**Keywords:** pregnant women, prevalence of COVID-19, relaxation of epidemic control measures, neonatal infections, risk factor

## Abstract

**Objective:**

To investigate the epidemiological characteristics of COVID-19 in pregnant women after relaxation of epidemic control measures.

**Methods:**

This cross-sectional study employed a multistage stratified sampling method, involving six sampling sites (districts/counties) of three cities (Zhuzhou, Chenzhou, and Huaihua) in Hunan Province, China. A questionnaire-based survey collected basic maternal information, COVID-19 infection status, and pregnancy-related information of the participants. Data were extracted and double checked for accuracy. Statistical analyses were conducted using SPSS 25.0 software.

**Results:**

Among the 7,761 pregnant women included in the study, 5,191 (66.9%) had a positive result of SARS-CoV-2 test or related symptoms. The majority of maternal infections were mild (90.0%), and a very small fraction were severe and critical (0.4% and 0.1 %). Headache and body aches (65.3%) were the most common symptoms. Of the 5,191 pregnant women with COVID-19, 4,150 (79.9%) had no complications during pregnancy. A total of 2,711 (52.2%) infected women had deliveries, and 449 (16.6%) newborns had infections. The impacts of COVID-19 on adverse pregnancy outcomes were limited. Logistic multivariable regression analysis showed that pregnant women with an education level of junior college and above (OR = 1.39, 95% CI: 1.18, 1.64), those with a monthly household income ≥ 3,000 yuan and above (OR = 1.18, 95% CI: 1.03, 1.34), those who lived with their family during family member infection (OR = 1.48, 95% CI: 1.32, 1.66), and those with pulmonary (OR = 1.41, 95% CI: 1.07, 1.85) or other (OR = 1.40, 95% CI: 1.19, 1.65) underlying diseases were more likely to have COVID-19. A farmer/worker occupation type (OR = 0.62, 95% CI: 0.48, 0.79) was a protective factor.

**Conclusion:**

A high prevalence of COVID-19 in pregnant women following relaxation of control measures has been observed at provincial scale in China. Most cases were mild, and few effects on newborns were observed. Higher education and income, living with infected family members, and having pulmonary disease were identified as risk factors, suggesting that mobility is the most critical factor influencing infection rates. This study provides useful references for epidemic prevention and control in the future.

## 1 Introduction

The outbreak of coronavirus disease 2019 (COVID-19), caused by severe acute respiratory syndrome coronavirus 2 (SARS-CoV-2) infection, was declared as a public health emergency by the World Health Organization (WHO) in March 2020 (1, 2). The pandemic had posed an unprecedented challenge to the public health system, registering over 700 million confirmed cases and an estimated 7 million fatalities globally by July 2023 (3). In China, strict prevention and control measures were adopted in response to the COVID-19 pandemic; and as of December 23rd, 2022, 3,97,195 confirmed cases and 5,241 deaths had been reported (4).

There has been conflicting literature regarding the risk of COVID-19 among pregnant women since the pandemic (5–7). A study from China reported that there was no increased risk of COVID-19 among pregnant women compared to the general population (6). In contrast, another study found that pregnant women had significantly higher rates of SARS-CoV-2 infection (7). The prevalence of asymptomatic infection in pregnant women was previously reported to be 54.1% (8). For pregnant women with COVID-19, the most common symptoms were fever (∼60%), cough (∼45%), and dyspnea (∼20%). Other symptoms included myalgia, fatigue, runny nose, chills, nausea and vomiting, rash, abdominal pain, dizziness, sore throat, nasal congestion and loss of appetite (9, 10). Maternal risk factors commonly associated with severe manifestations of COVID-19 included advanced age, gestational diabetes mellitus (GDM), chronic hypertension, asthma, high BMI, and preexisting cardiovascular disease. Some studies have shown that pregnancy may accelerate the clinical course of COVID-19. Compared with non-pregnant women, pregnant women with COVID-19 were at an increased risk of serious diseases, with increased rates of hospitalization, intensive care unit (ICU) admission, intubation and mortality (11, 12). COVID-19 has also been associated with adverse pregnancy outcomes. The most commonly reported pregnancy complications linked to COVID-19 were preterm birth (13, 14), premature rupture of membrane, preeclampsia, unreliable fetal detection or fetal distress, and stillbirths (15, 16).

With a nation-wide implementation of relaxation polices for COVID-19 during early December 2022 in China, a new wave of infection was implied, and self-management of symptoms and home observation were recommended for the general population. However, few studies have described the epidemiological characteristics of COVID-19 in pregnant women after relaxation of control measures.

This cross-sectional study was conducted to investigate COVID-19 infection in pregnant women after relaxation of control measures in Hunan province, China, with the aim to provide a scientific basis for epidemic prevention and control.

## 2 Materials and methods

### 2.1 Ethic approval

The study was approved by the Ethics Committee of Hunan Provincial Maternal and Child Health Care Hospital (No. 2021-S056). Written informed consent was obtained from all participants of the study.

### 2.2 Study population and sampling

This study investigated COVID-19 infection in pregnant women from 8 December 2022 to 18 March 2023 after relaxation of control measures in Hunan Province, China. During the time period, women in pregnancy or within 42 days after delivery were recruited. Multistage stratified sampling method was used in this study, and the questionnaire survey covered six districts/counties of three cities in Hunan Province. The geographic regions of Hunan Province were classified as economically developed, economically medium, and economically underdeveloped according to their economic situation (17). One city was selected at each of the three economic levels. Further, one urban district and one county were selected in each city for whole-group sampling. According to the cross-sectional survey formula $n=\frac{Z_{\text{α}/2}^{2}\text{π(}1-\text{π)}}{{\text{δ}}^{2}}(Z_{\text{α α}}=1.96)$, based on a COVID-19 infection rate of 60% through the outpatient pre-survey among pregnant women, the calculated minimum sample size for each sampling site was 1,025 women. An additional sampling of 20% of the calculated sample size was adopted to prevent sample loss. Thus, at least 1,230 women should be surveyed for each sampling site, and the minimum sample size for this study was 7,380 women.

### 2.3 Data collection

The questionnaire, which was developed by literature searches, policy evaluation, and expert consultation, mainly included the three following aspects: (1) basic maternal information (age, occupation type, education level, underlying diseases, etc.); (2) COVID-19 infection status (diagnosis method, symptoms, clinical subtype, etc.); and (3) information related to pregnancy (week of delivery, fetal weight, sequelae, etc.). The survey was conducted by trained investigators, and data quality checks were carried out twice per week. The survey was completed by the respondents filling in the form online. In this study, pregnant women who had clinical symptoms without a hospital diagnosis were included as COVID-19-positive patients, considering a concentrated outbreak of infection and an excessive burden of medical testing for COVID-19 in the short period after relaxation of control measures in China.

### 2.4 Statistical analysis

Descriptive analyses were carried out using frequencies and percentages for categorical variables and medians and quartiles for non-normal continuous variables. Pearson chi-square test for single factor analysis was performed to identify statistically significant associations between COVID-19 infection and categorical explanatory variables. Multivariable logistic regression was used to control for confounding variables and obtain the odds ratios for each explanatory variable and the respective 95% confidence intervals (CIs) and *p*-values. A two-sided *p* < 0.05 was considered statistically significant. Statistical analyses were conducted using SPSS 25.0 software (IBM SPSS Inc., Chicago, IL, United States).

## 3 Results

### 3.1 Socio-demographic and disease characteristics

**FIGURE 1:**
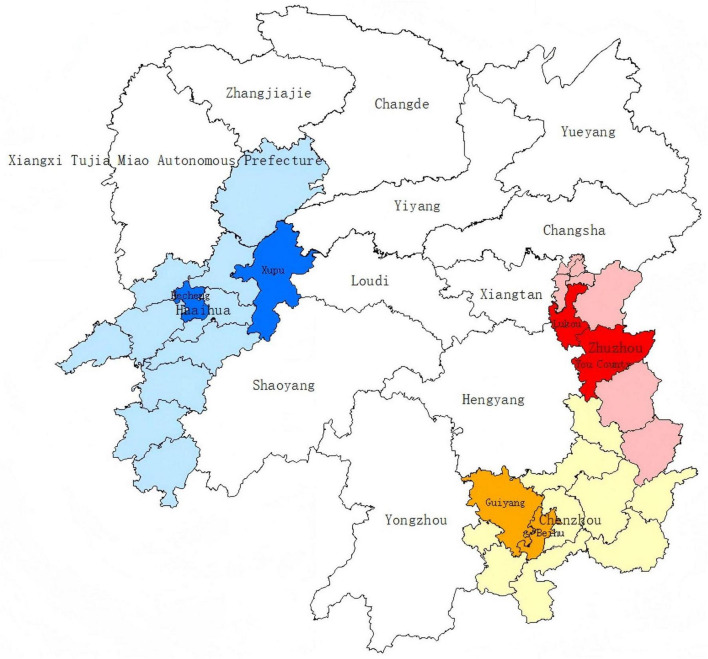
Six sampling sites of three cities in Hunan Province, China, in a geographic information system (GIS). The sampling sites include Lukou District and You County affiliated to economically developed Zhuzhou City, Beihu District and Guiyang County affiliated to economically medium Chenzhou City, and Hecheng District Xupu County affiliated to economically underdeveloped Huaihua City.

A total of 7,841 pregnant women were recruited from six sampling sites of three cities in Hunan Province, China (Figure 1). After a quality check, the questionnaires of 80 participants did not meet the eligibility criteria. The final analysis included 7,761 participants, with an age range of 15–49 years. Of all the participants, 5,191 (66.9%) had a positive result of SARS-CoV-2 test or related symptoms. Comparing the COVID19 group with the non-COVID19 group, single factor analysis showed that pregnant women who lived in urban areas, had an education level of junior college or higher, had an occupational status of business owner/freelancer/unemployed, had a monthly household income ≥ 5,000 yuan, lived with their families during family member infection, or had other underlying diseases were more likely to be infected (*P* < 0.001) (Table 1).

**TABLE 1:** Demographic and disease characteristics of study participants by COVID-19 status.

Variable	Total (*n* = 7,761)	COVID-19		*P*-value*
		Yes (*n* = 5,191)	No (*n* = 2,570)	
**Age group, years**				0.079
15–24	1,523 (19.6)	986 (19.0)	537 (20.9)	–
25–34	4,795 (61.8)	3,249 (62.6)	1,546 (60.2)	–
35–49	1,443 (18.6)	956 (18.4)	487 (18.9)	–
**Area**				< 0.001
Urban	1,456 (18.8)	1,038 (20.0)	418 (16.3)	–
Rural	6,305 (81.2)	4,153 (80.0)	2,152 (83.7)	–
**Ethnicity**				0.096
Han	7,263 (93.6)	4,841 (93.3)	2,422 (94.2)	–
Others	498 (6.4)	350 (6.7)	148 (5.8)	–
**Education level**				< 0.001
Junior high school and below	2,985 (38.5)	1,840 (35.4)	1,145 (44.6)	–
Senior high school	2,481 (32.0)	1,604 (30.9)	877 (34.1)	–
Junior college and above	2,295 (29.6)	1,747 (33.7)	548 (21.3)	–
**Occupational status**				< 0.001
Staff member	1,972 (25.4)	1,119 (21.6)	853 (33.2)	–
Farmer/worker	1,802 (23.2)	1,239 (23.9)	563 (21.9)	–
Business owner/freelancer	876 (11.3)	680 (13.1)	196 (7.6)	–
Unemployed	1,698 (21.9)	1,216 (23.4)	482 (18.8)	–
Others	1,413 (18.2)	937 (18.1)	476 (18.5)	–
**Monthly household income, yuan**				< 0.001
< 3,000	2,738 (35.3)	1,633 (31.5)	1,105 (43.0)	–
3,000–4,999	2,369 (30.5)	1,599 (30.8)	770 (30.0)	–
≥ 5,000	2,654 (34.2)	1,959 (37.7)	695 (27.0)	–
**Residential status of subjects during household member infection**				< 0.001
Living together	3,079 (39.7)	2,237 (43.1)	842 (32.8)	–
Not living together	4,682 (60.3)	2,954 (56.9)	1,728 (67.2)	–
**Underlying pulmonary disease** ^#^				0.060
Yes	289 (4.6)	209 (5.0)	80 (3.9)	–
No	5,934 (95.4)	3,976 (95.0)	1,958 (96.1)	–
**Other underlying diseases**				< 0.001
Yes	1,108 (14.3)	816 (15.7)	292 (11.4)	–
No	6,653 (85.7)	4,375 (84.3)	2,278 (88.6)	–

Data are shown in the form of *n* (%).

*Chi-square test or Fisher’s exact test.

^#^A total of 1,538 out of the 7,761 participants did not provide information of underlying pulmonary disease.

### 3.2 Disease status of pregnant women with COVID-19

**FIGURE 2:**
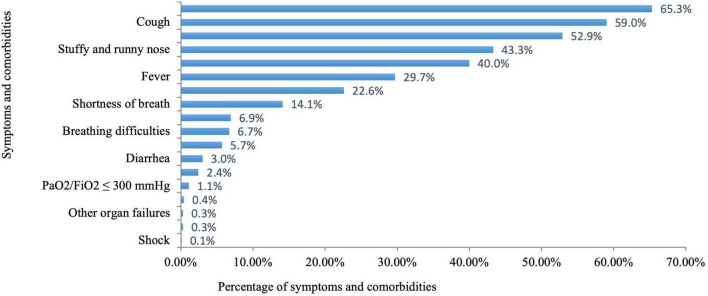
Percentage of symptoms and comorbidities in pregnant women with COVID-19.

As shown in Figure 2 and Table 2, among the 5,191 pregnant women with infections, 2,171 (41.8%) had a positive result of SARS-CoV-2 test. The majority of pregnant women had mild infections (90.0%), and a very small fraction had severe and critical infections (0.4 and 0.1%). Headache and body aches (65.3%) were the most common symptoms in infected pregnant women, followed by cough (59.0%), physical weakness (52.9%), stuffy and runny nose (43.3%), fever (29.7%), and a decreased sense of taste or smell (22.6%). These symptoms showed varying duration as shown in Table 2. The least common comorbidities in pregnant women with COVID-19 included shock (0.1%), respiratory failure (0.3%), other organ failure (0.3%), and progressive clinical symptoms (0.4%). Among the 5,191 infected pregnant women, 1,930 (52.9%) used antipyretic drugs for fever; 1,719 (47.1%) did not take antipyretic drugs due to either fear of adverse effects on the fetus (*n* = 1,383, 37.9%) or unavailability of the drugs (*n* = 336, 9.2%). A total of 1,720 (33.1%) infected pregnant women sought medical advice. Of these patients, 154 (3.0%) were diagnosed with pneumonia by physicians; 405 (7.8%) were hospitalized; and 35 (0.7%) needed ICU monitoring.

**TABLE 2:** Disease status of COVID-19-infected pregnant women in Hunan Province.

Disease status	*n* (%)/median (IQR)
**Diagnosis method of COVID-19**	
Positive immunological test	2,171 (41.8)
Not tested, but flu-like symptoms	3,020 (58.2)
**Duration of symptoms, days**	
Fever	2 (1–3)
Headache or body aches	2 (2–3)
Physical weakness	3 (2–5)
Cough	7 (5–15)
Stuffy and runny nose	5 (3–8)
Dry and sore throat	5 (3–7)
Vomiting	2 (1–3)
Diarrhea	2 (1–3)
Decreased sense of taste or smell	8 (5–15)
Breathing difficulties	5 (2–7)
Other symptoms	3 (1–7)
**Use of antipyretics for fever (*n* = 3,649)**	
Yes	1,930 (52.9)
No: fear of adverse effects on the fetus	1,383 (37.9)
No: unavailability of antipyretics due to insufficient supply	336 (9.2)
Type of infection*	
Mild	4,672 (90.0)
Moderate	496 (9.6)
Severe	19 (0.4)
Critical	4 (0.1)
**Sought medical advice**	
Yes	1,720 (33.1)
No	3,471 (66.9)
**Inpatient treatment**	
Yes	405 (7.8)
No	4,786 (92.2)
**ICU monitoring**	
Yes	35 (0.7)
No	5,156 (99.3)
**Diagnosis of pneumonia by doctors**	
Yes	154 (3.0)
No	5,037 (97.0)

Total *n* = 5,191, unless stated separately.

*The classification of COVID-19 infection types is based on the Treatment Plan for Novel Coronavirus Infection (10th Trial Version) by China’s National Health Commission (NHC).

### 3.3 Pregnancy and delivery status of pregnant women with COVID-19

Of the 5,191 pregnant women with COVID-19, 4,150 (79.9%) had no complications during pregnancy, while 128 (2.5%) had an abnormal heart rate, and 72 (1.4%) had abnormal vaginal bleeding, and 606 (11.7%) had other complications. With respect to the 2,711 (52.2%) pregnant women with COVID-19 who had deliveries, the median gestational age was 38 (IQR 37, 39) weeks, while the rate of cesarean birth was 20.7%, and the median neonatal weight was 3,200 (IQR 2980, 3,500) g; and 449 (16.6%) newborns had infections. A total of 470 (17.7%) pregnant women with COVID-19 presented with decreased breast milk production after delivery, while 168 (6.2%) presented with persistent lochia and 64 (2.4%) presented with fever lasting more than 5 days, and 6 (0.2%) presented with abnormal vaginal bleeding after delivery (Table 3).

**TABLE 3:** Pregnancy and delivery status of COVID-19-infected pregnant women.

Pregnancy and delivery status	*n* (%)/median (IQR)
Complications during pregnancy after infection (*n* = 5,191)	32 (0.6)
Early pregnancy abortion	36 (0.7)
Fetal intrauterine distress	25 (0.5)
Premature rupture of membranes	60 (1.2)
Preterm birth	10 (0.2)
Abnormal vaginal bleeding	72 (1.4)
Abnormal blood pressure	22 (0.4)
Abnormal heart rate	128 (2.5)
Abnormal blood sugar	50 (1.0)
Others	606 (11.7)
No complications	4,150 (79.9)
**Delivery method (*n* = 5,191)**	
Natural birth	1,637 (31.5)
Cesarean birth	1,074 (20.7)
Undelivered	2,480 (47.8)
**Gestational week, weeks**	38 (37, 39)
Neonatal weight, g	3,200 (2,980, 3,500)
**Neonatal infection**	
Yes	449 (16.6)
No	2,262 (83.4)
**Complications after birth**	
Decreased breast milk production	470 (17.3)
Persistent lochia	168 (6.2)
Vaginal bleeding	6 (0.2)
Persistent fever for more than 5 days	64 (2.4)
No abnormalities	2,003 (73.9)

*n* = 2,711, unless stated separately.

### 3.4 Factors influencing maternal infection with COVID-19

The results of the logistic multivariable regression analysis are presented in Table 4. Education level, occupational status, monthly household income, residential status during household member infection, and underlying disease were factors influencing maternal COVID-19 infection. Specifically, pregnant women with an education level of junior college and above (OR = 1.39, 95% CI: 1.18, 1.64), those with a monthly household income ≥ 3,000 yuan and above (OR = 1.175, 95% CI: 1.03, 1.34), those who lived with their family during family member infection (OR = 1.48, 95% CI: 1.32, 1.66), and those with pulmonary (OR = 1.41, 95% CI: 1.07, 1.85) or other (OR = 1.40, 95% CI: 1.19, 1.65) underlying diseases were more likely to have COVID-19. A farmer/worker (OR = 0.62, 95% CI: 0.48, 0.79) and other (OR = 0.72, 95% CI: 0.57, 0.91) occupation types were protective factors.

**TABLE 4:** Multivariable logistic regression of factors associated with COVID-19 infection among pregnant women.

Variable	β	SE	Wald’s test	*P*-value	OR (95% CI)
**Education level**					
Junior high school and below	–	–	–	–	Ref
Senior high school	-0.05	0.07	0.57	0.45	0.95 (0.84, 1.08)
Junior college and above	0.33	0.08	15.74	0.00	1.39 (1.18, 1.64)
**Occupational status**					
Staff member	–	–	–	–	Ref
Farmer/worker	-0.48	0.12	15.31	0.00	0.62 (0.48, 0.79)
Business owner/freelancer	-0.19	0.12	2.45	0.12	0.831 (0.67, 1.05)
Unemployed	-0.05	0.12	0.18	0.67	0.95 (0.75, 1.21)
Others	-0.33	0.12	7.36	0.01	0.72 (0.57, 0.91)
**Monthly household income, yuan**					
< 3,000	–	–	–	–	Ref
3,000–4,999	0.16	0.07	5.48	0.02	1.18 (1.03, 1.34)
≥ 5,000	0.49	0.07	45.80	0.00	1.62 (1.41, 1.87)
**Residential status of subjects after household member infection**					
Not living together	–	–	–	–	Ref
Living together	0.39	0.06	44.34	0.00	1.48 (1.32, 1.66)
**Underlying pulmonary disease**					
No	–	–	–	–	Ref
Yes	0.34	0.14	5.97	0.02	1.41 (1.07, 1.85)
**Other underlying diseases**					
No	–	–	–	–	Ref
Yes	0.34	0.08	16.26	0.00	1.40 (1.19, 1.65)

## 4 Discussion

The present study investigated the epidemiological characteristics of COVID-19 in pregnant women in a rapid spread of infection after relaxation of control measures in Hunan Province, China. A high prevalence (66.9%) of COVID-19 among pregnant women was observed in this study. A previous study in sub-Saharan Africa showed that 36.9% of participants had a positive result of SARS-CoV-2 test (3). Another hospital-based study in the USA reported a COVID-19 prevalence of 8.0% in pregnant women (18). Apparently, the infection rate in our study was much higher than those observed in other studies. This can be mainly attributed to the varying control measures in different countries. During the global pandemic, a low profile of COVID was maintained in China under strict prevention and control measures. This cross-sectional study fell into a new wave of infection after relaxation of control measures, and a high prevalence of COVID-19 was expected to the whole population in the country during the short time period. The second reason for a high infection rate in pregnant women can be that this study included those who had clinical symptoms but were not diagnosed at hospital.

A previous study indicated that pregnant women who possess a secondary level of education are less susceptible to contracting COVID-19 (19). In this study, pregnant women with a college education or above were more likely to be infected. This finding is in line with a previous result that higher income individuals are more susceptible to infection (20). It is possible that a higher level of social engagement increases the risk of infection for well-educated pregnant women. The study of Chung et al. (21) showed that human mobility can accelerate the spread of COVID-19. The current study demonstrates that living in urban areas, working as a business owner/freelancer, having a monthly household income ≥ 5,000 yuan, and living with infected family members are associated with a higher risk of infection. Therefore, it can be inferred that the movement of people is the biggest risk factor for infection, which is consistent with the findings in other studies from China (19).

Additionally, this study shows that pregnant women with pulmonary (OR = 1.408) or other (OR = 1.398) underlying diseases are more likely to have COVID-19 infection. COVID-19 is transmitted predominantly through the airways and is highly related to the human immune system (14, 22). The study of Kato et al. (23) showed SARS-CoV-2 infection was associated with a high prevalence of distal airspace mucus accumulation and increased MUC5B expression in COVID-19 autopsy lungs. Early data suggest that potentially severe long-term consequence of COVID-19 is development of long COVID-19-related interstitial lung disease (24). Therefore, people with pulmonary or other underlying diseases were at higher risk of infection.

The current study has found that most maternal infections had mild symptoms, whereas only a small fraction had severe and critical (0.4 and 0.1%) manifestations. This rate was much lower than that of severe cases or cases resulting in death in a previous meta-analysis (12%) (8). A meta-analysis showed that pregnant women with COVID-19 had increased odds of death (odds ratio = 6.09) compared to pregnant women without COVID-19 (13). The study of Edlow et al. (25) showed that among pregnant women with SARS-CoV-2 infection, 11% had moderate disease, 16% had severe disease, and 3% had critical disease. The lower rates of severe and critical cases in this study might be explained by the weakened pathogenicity and virulence of SARS-CoV-2 variants in the final stages of the pandemic. Based on existing reports (7–10), most cases of COVID-19 in pregnant women occurred in the third trimester of pregnancy. By contrast, this study shows that it can occur in all stages of pregnancy. This difference may result from the distinct sampling strategies between the studies, namely hospital-based investigation versus population-based questionnaire survey. In the previous hospital-based studies, women in the third trimester of pregnancy were more likely to be admitted to hospital because of a need for medical care, whereas this study was orientated to a broader population.

In this study, 2,711 infected women had deliveries, of whom 449 (16.56%) had neonates with infections. This increased proportion of postnatal infections may be related to the increased risk of exposure to the virus after the full relaxation of control measures. Until now, there has been no evidence that pregnant women with SARS-CoV-2 are at risk of mother-to-child transmission (25, 26). In a study with a collection of 88 placenta samples, SARS-CoV-2 RNA was not detected. It has also been shown that the mother-to-neonate transfer of anti-SARS-CoV-2 antibodies was significantly lower than that of anti-influenza hemagglutinin A antibodies (20), and that there were no placental pathological differences by illness severity of SARS-CoV-2 infection in pregnant women (27).

Furthermore, this study showed that 2,711 infected pregnant women delivered with a median gestational age of 38 weeks, a cesarean birth rate of 20.7%, and a median neonatal weight of 3,200 g. These data are consistent with the findings in a previous observation among 3,98,368 pregnant women from the general population in Hunan province (28), where cesarean section rate, mean birth weight, and mean gestational week at birth were 26.98%, 3,284.84 ± 372.04 g, and 38.94 ± 1.67 weeks, respectively. Therefore, relaxation of COVID-19 control measures in China did not result in increased adverse pregnancy outcomes.

However, there were limitations in the study. First, pregnant women who had symptoms but were not diagnosed at hospital were counted as COVID-19 taking into account of a concentrated outbreak of infection and an excessive burden of medical testing for COVID-19 at the special time window. A very small faction of pregnant women with a common cold might be counted as having COVID-19 in our study. Second, our survey was a retrospective study that may have recall information bias. Therefore, trained investigators and regular logic checks to reduce bias are important for data quality.

In conclusion, this study has described the profile of COVID-19 infection in pregnant women and the impact on pregnancy outcomes at provincial scale after nation-wide relaxation of epidemic control measures. Despite a high prevalence of COVID-19 among pregnant women, most cases were mild, and few adverse effects on pregnancy outcomes were observed. Mobility is the most critical factor influencing infection rates. It is expected that the findings provide useful references for epidemic prevention and control in the future.

## Data Availability

The raw data supporting the conclusions of this article will be made available by the authors, without undue reservation.
